# Investigating the Association of Subjective Numeracy, Interpersonal Communication, and Perceived Discrimination With Watching Health-Related Videos on Social Media Platforms: Cross-Sectional Analysis

**DOI:** 10.2196/71971

**Published:** 2025-11-05

**Authors:** Katerina Andreadis, Nancy Buderer, Aisha Langford

**Affiliations:** 1 Department of Population Health, NYU Grossman School of Medicine, 227 E 30th Street, 6th floor, New York, NY, 10016, United States, 1 9175555568; 2 Nancy Buderer Consulting, LLC, Oak Harbor, OH, United States; 3 Department of Family Medicine and Public Health Sciences, Wayne State University, Detroit, MI, United States

**Keywords:** social media, health communication, perceived discrimination, information dissemination, health-related videos

## Abstract

**Background:**

Over the past two decades, use of social media has grown among US adults. Common social media platforms include Facebook, YouTube, Instagram, X, LinkedIn, and TikTok. People proactively use social media for a variety of purposes including searching for health information, peer-to-peer social support, and health-related information sharing. As these platforms often serve as sources of health information, understanding how, if at all, people use them may inform future behavioral interventions delivered via social media. Additionally, a better understanding of social engagement may have implications for public health messaging and patient-centered communication.

**Objective:**

Using a nationally representative sample of US adults, we explored how factors including subjective numeracy (ie, ease of understanding medical statistics), interpersonal communication with family and friends, and perceived discrimination influence whether people ever watched versus never watched health-related videos on social media platforms.

**Methods:**

We analyzed the National Cancer Institute’s Health Information National Trends Survey data, which were collected from March to November 2022 (n=6252). After excluding participants who did not have complete data for all variables of interest, we analyzed responses from 4543 participants. Respondents were asked, “In the past 12 months, how often did you watch a health-related video on a social media site (eg, YouTube)?” Response options included: almost every day, at least once a week, a few times a month, less than once a month, and never. We collapsed answers into ever or never watched. Odds ratios (OR), 95% CIs, and *P* values were calculated. A multivariate logistic regression model was considered using all factors that were univariately significant (*P*<.10). Using backward elimination, factors that were not significant with *P*>.05 were removed one by one until remaining factors were all significant collectively (*P*<.05).

**Results:**

Of 4543 adults analyzed, 61.5% reported watching at least one health-related video in the past 12 months, whereas 38.5% had never watched one. In the multivariable analysis, all age group categories over 50 years were less likely to watch health-related videos compared to those aged 18‐34 years, with respondents aged ≥75 years having the lowest odds of all groups for watching a health-related video (OR 0.16, *P*<.001). Higher odds of watching health-related videos were observed among respondents who were Black (OR 1.59, *P*<.01), Hispanic (OR 1.54, *P*=.01), and from “Other” minority groups (OR 2.07, *P*=.01) compared to White respondents. College graduates (OR 1.71, *P*<.01) and those who found medical statistics easy to understand (OR 1.29, *P*=.04), talked about health with friends or family (OR 1.68, *P*<.01), or experienced racial discrimination in medical care (OR 1.59, *P*=.02) also had higher odds of watching health-related videos on social media.

**Conclusions:**

Findings from this study may help target health communication campaigns on social media designed to improve screening, lifestyle changes, medication adherence, and disease management.

## Introduction

In 2023, eight in ten US adults engaged with platforms like YouTube, nearly half of US adults used Instagram, and over one-third of US adults used TikTok [1]. Health information is a major component of this engagement, with 85% of adults seeking health information on social media and nearly 40% watching health-related YouTube videos [2,3]. Since 2018, social media has overtaken traditional print news as the primary source of news and continues to expand its influence on public perceptions and knowledge about health [4].

The internet has allowed for anyone to disseminate health information, which has both benefits and drawbacks [5]. On the one hand, patients and community groups can share helpful information without going through gatekeepers. Online health communities enable peer-to-peer support and patient-generated health information sharing, providing emotional support and experiential knowledge that complements traditional health care [6]. On the other hand, democratization and ease of information sharing may increase the quantity of misinformative and inaccurate health information on social media platforms. Systematic reviews have documented a substantial prevalence of health misinformation across social media platforms, with studies finding significant rates of inaccurate health content on X (formerly Twitter), YouTube, and Facebook [7,8].

In recent years, a growing number of health care professionals and health systems have been sharing health-related content on social media [9-11]. A systematic review in 2023 found that researchers and clinicians use social media to facilitate research, for professional development, and to enhance patient-provider communication and offline services (eg, medication adherence), while the public uses it to seek and share health information, exchange social support, and document their health experiences [11]. Professional guidelines on the responsible use of social media advise clinicians to keep personal and professional identities separate and to protect patient privacy and confidentiality online [12]. Although posts about medical tests from physicians are more likely to mention harms and less likely to have an overall promotional tone, health misinformation remains prevalent, with most posts being misleading, not reporting medical harms, or not citing scientific evidence [13]. In a newer analysis of TikTok transcripts on diabetic foot care, 42% of posts contained misleading or false claims, with advice that could delay treatment or worsen outcomes [14]. Finally, algorithmic recommendation systems shape what health content people see: audits of YouTube report that recommendations are conditioned by users’ watch histories, and analyses of X have observed that posts from influential accounts with high engagement, including low-credibility content, receive increased visibility [15,16]. These trends highlight the need to identify which populations engage with health-related video content on social media.

Accordingly, this exploratory study focuses on how various sociodemographic and health-related variables influence engagement with health-related videos on social media platforms. By understanding these factors, we aim to provide contemporary data on the use of social media for health-related purposes, particularly for populations who may use social media more often.

## Methods

### Data Sources

This study used self-reported data from the Health Information National Trends Survey (HINTS 6), collected in 2022. HINTS collects data from a nationally representative sample of the US population, focusing on trends in health communication and health information technology. The HINTS 6 dataset originally included 6252 respondents, who completed the survey online or by paper [17].

### Variables of Interest

#### Main Outcome

Participants were asked, “In the past 12 months, how often did you watch a health-related video on a social media site (for example, YouTube)?” Responses were combined into two groups: *ever watched* (a combination of almost every day, at least once a week, a few times a month, less than once a month) and *never watched*.

#### Sociodemographic and Health-Related Covariates

We collected sociodemographic and health-related information including age, birth gender, race/ethnicity, education level, self-rated general health, BMI, and history of chronic conditions such as diabetes, high blood pressure, depression, and cancer. Additional covariates included confidence in completing medical forms, confidence in taking care of one’s health, confidence in finding health resources online, ease of understanding medical statistics, discussions about health with friends and family, trust in health care systems, and experiences of perceived racial discrimination when obtaining medical care.

### Statistical Methods

Odds ratios (OR), 95% CIs, and *P* values were calculated using SAS (Statistical Analysis System) proc surveylogistic with jackknife weighting, including the overall weight and 50 replicate weights, and the Newton-Raphson algorithm [17]. Results are presented as the OR (95% CI) for the odds of yes (ever watched video) and *P* value. When interpreting ORs, confidence intervals that do not include the value 1 were considered significant. Data were analyzed using SAS (version 9.4; SAS Institute Inc) and SAS/STAT (version 14.1; SAS Institute Inc). HINTS 6 contained 6252 responses. Of these, there were 6158 with a response to the primary question of interest. We further eliminated respondents if they were missing data on any of the variables in the study. Therefore, analyses are based on a sample size of 4543.

### Ethical Considerations

The HINTS 6 data collection protocol received institutional review board (IRB) approval by the Westat IRB (project number 6632.03.51) [18]. This secondary analysis study used only publicly available, deidentified data from the HINTS 6 survey, and therefore did not undergo IRB review as it fits the secondary research exemption under federal guidelines (45 Code of Federal Regulations 46.104) [18]. Data were accessed under the HINTS public use policy, with no additional permissions required.

## Results

As shown in Table 1, 61.5% of respondents watched a health-related video on a social media website in the past 12 months, while 38.5% never watched a video. In the multivariable analysis (Table 2), respondents aged 50 years and older were less likely to watch health-related videos compared to those aged 18‐34 years. Specifically, compared to those aged 18‐24 years, those aged 50‐64 years had an OR of 0.58 (95% CI 0.38‐0.88); those aged 65‐74 had an OR of 0.28 (95% CI 0.17‐0.45), and those aged 75 and older had an OR of 0.16 (95% CI 0.10‐0.25).

**Table 1.** Univariable associations with watching health-related videos on social media, with data from the Health Information National Trends Survey 6, United States, 2022 (N=4543).^a^

	Watched (n=2671, 61.5%), % of row (SE)^b^	Never watched (n=1872, 38.5%), % of row (SE)	Odds ratio for Watched (95% CI)	*P* value
Age (mean years)	45.8 (0.45)	55.7 (0.83)	0.97 (0.96-0.97)	<.001
Age group				<.001
18‐34	74.3 (3.5)	25.7 (3.5)	Ref^c^	
35‐49	70.7 (2.8)	29.3 (2.8)	0.84 (0.50-1.40)	.49
50‐64	61.3 (2.3)	38.7 (2.3)	0.55 (0.37-0.81)	.003
65‐74	42.9 (2.2)	57.1 (2.2)	0.26 (0.17-0.41)	<.001
≥75	28.5 (2.6)	71.5 (2.6)	0.14 (0.09-0.22)	<.001
Birth gender				
Female	63.0 (1.4)	37.0 (1.4)	1.15 (0.94-1.40)	.17
Male	59.7 (2.0)	40.3 (2.0)	Ref	
Race/ethnicity				<.001
Non-Hispanic White	56.5 (1.6)	43.5 (1.6)	Ref	
Non-Hispanic Black or African American	68.1 (3.1)	31.9 (3.1)	1.65 (1.24-2.19)	.001
Hispanic	69.0 (2.6)	31.0 (2.6)	1.72 (1.29-2.28)	<.001
Non-Hispanic Asian	77.3 (5.0)	22.7 (5.0)	2.62 (1.43-4.82)	.003
Non-Hispanic other	77.1 (4.0)	22.9 (4.0)	2.60 (1.60-4.21)	<.001
Education				
College graduate	70.2 (1.4)	29.8 (1.4)	1.80 (1.47-2.19)	<.001
Not college graduate	56.8 (1.8)	43.2 (1.8)	Ref	
General health				.21
Excellent or very good	61.2 (2.1)	38.8 (2.1)	1.20 (0.83-1.74)	.32
Good	63.9 (1.9)	36.1 (1.9)	1.35 (0.95-1.90)	.09
Fair or poor	56.8 (3.7)	43.2 (3.7)	Ref	
Diabetes				
Yes	56.5 (3.3)	43.5 (3.3)	0.78 (0.59-1.03)	.08
No	62.5 (1.3)	37.5 (1.3)	Ref	
High blood pressure				
Yes	55.0 (2.1)	45.0 (2.1)	0.65 (0.54-0.78)	<.001
No	65.4 (1.3)	34.6 (1.3)	Ref	
Heart condition				
Yes	46.4 (4.3)	53.6 (4.3)	0.51 (0.36-0.73)	<.001
No	62.7 (1.3)	37.3 (1.3)	Ref	
Lung disease				
Yes	59.5 (3.3)	40.5 (3.3)	0.91 (0.66-1.25)	.55
No	61.8 (1.4)	38.2 (1.4)	Ref	
Depression				
Yes	67.6 (1.9)	32.4 (1.9)	1.47 (1.18-1.82)	<.001
No	58.7 (1.6)	41.3 (1.6)	Ref	
Ever had cancer				
Yes	50.3 (2.8)	49.7 (2.8)	0.60 (0.47-0.77)	<.001
No	62.8 (1.3)	37.2 (1.3)	Ref	
BMI group				.37
Underweight or normal weight (<25)	60.9 (2.3)	39.1 (2.3)	1.06 (0.81-1.39)	.65
Overweight (25‐29.9)	64.1 (2.3)	35.9 (2.3)	1.22 (0.92-1.60)	.16
Obese (>30)	59.5 (2.2)	40.5 (2.2)	Ref	
Confident with filling out medical forms				
Very	63.2 (1.7)	36.8 (1.7)	1.20 (0.97-1.49)	.10
Somewhat, a little, not at all	58.9 (1.9)	41.1 (1.9)	Ref	
Confident to take good care of your health				
Completely	57.1 (2.6)	42.9 (2.6)	0.78 (0.63-0.97)	.03
Very, somewhat, a little, not at all	63.1 (1.2)	36.9 (1.2)	Ref	
Confident to find internet health resources				
Completely	74.2 (4.0)	25.8 (4.0)	1.96 (1.24-3.09)	.005
Very, somewhat, a little, not at all	59.5 (1.4)	40.5 (1.4)	Ref	
Understand medical statistics				
Very easy, easy	63.4 (1.4)	36.6 (1.4)	1.38 (1.09-1.75)	.01
Hard, very hard	55.6 (2.6)	44.4 (2.6)	Ref	
Patient-centered communication scale (mean linearized score on a 0‐100 scale)	76.1 (0.52)	78.5 (0.85)	1.00 (0.99-1.00)	.04
Talk about health with friends				.01
Yes	63.1 (1.3)	36.9 (1.3)	1.49 (1.10-2.01)	
No	53.5 (3.5)	46.5 (3.5)	Ref	
Trust the health care system				
Very	58.6 (2.0)	41.4 (2.0)	0.82 (0.69-0.98)	.03
Somewhat, a little, not at all	63.3 (1.3)	36.7 (1.3)	Ref	
Racially discriminated against when getting medical care				
Yes	77.5 (3.3)	22.5 (3.3)	2.26 (1.50-3.40)	<.001
No	60.3 (1.3)	39.7 (1.3)	Ref	

a Cross-sectional, survey-weighted logistic regressions estimating unadjusted odds ratios with 95% CIs for “ever watched” (≥1 time in past 12 mo) versus “never” in response to the question “In the past 12 months, how often did you watch a health-related video on a social media site (for example, YouTube)?” Row percentages are weighted.

b SE: weighted standard error.

c Ref: reference category of the independent variable.

**Table 2.** Multivariable associations with watching health-related videos on social media, with data from the Health Information National Trends Survey 6, United States, 2022.^a^

	Adjusted odds ratio for Watched versus Never (95% CI)	*P* value
Age group		<.001
18‐34	Ref	
35‐49	0.82 (0.50-1.35)	.43
50‐64	0.58 (0.38-0.88)	.01
65‐74	0.28 (0.17-0.45)	<.001
≥75	0.16 (0.10-0.25)	<.001
Race/ethnicity		.001
Non-Hispanic White	Ref	
Non-Hispanic Black or African American	1.59 (1.19-2.23)	.002
Hispanic	1.54 (1.11-2.11)	.01
Non-Hispanic Asian	1.72 (0.90-3.25)	.10
Non-Hispanic other	2.07 (1.19-3.60)	.01
Education		
College graduate	1.71 (1.36-2.16)	<.001
Not college graduate	Ref	
Confident to take good care of your health		
Completely	0.71 (0.57-0.89)	.004
Very, somewhat, a little, not at all	Ref	
Understand medical statistics		
Very easy, easy	1.29 (1.01-1.65)	.04
Hard, very hard	Ref	
Talk about health with friends		
Yes	1.68 (1.23-2.31)	.002
No	Ref	
Racially discriminated against when getting medical care		
Yes	1.59 (1.08-2.34)	.02
No	Ref	

a Survey-weighted logistic regression modeling the odds of “ever watched” (≥1 time in past 12 months mo) versus “never” in response to the question “In the past 12 months, how often did you watch a health-related video on a social media site (for example, YouTube)?“ Factors that were univariately significant with *P*<.10 were attempted in the model. Using backward elimination, factors were removed one by one until all of the remaining factors in the model were significant with *P*<.05. Adjusted odds ratios with 95% CIs are shown, reference categories are indicated by “Ref,” and overall *P* values are reported for multilevel predictors.

Respondents who identified as Black (OR 1.59, 95% CI 1.19‐2.23), Hispanic (OR 1.54, 95% CI 1.11‐2.11), and “Other” races (OR 2.07, 95% CI 1.19‐3.60) were more likely to have watched health-related videos compared to White respondents. College graduates (OR 1.71, 95% CI 1.36‐2.16), those who found medical statistics easy to understand (OR 1.29, 95% CI 1.01‐1.65), those who discussed health with friends and family (OR 1.68, 95% CI 1.23‐2.31), and those who experienced racial discrimination in health care settings (OR 1.59, 95% CI 1.08‐2.34) also had higher odds of watching health-related videos. Conversely, respondents who were “completely confident” in their ability to take good care of their health were less likely to watch health-related videos on social media (OR 0.71, 95% CI 0.57‐0.89).

## Discussion

### Principal Findings

The purpose of this exploratory study was to examine the associations between various sociodemographic and health-related factors and watching health-related videos on social media platforms. Our results indicate that age, race/ethnicity, education, subjective numeracy, interpersonal communication, and perceived discrimination are significantly associated with watching health-related videos on social media.

### Comparison With Prior Work

To date, several studies have used HINTS data to explore different aspects of health information–seeking and communication, including watching health-related videos on social media platforms [19,20]. For example, Garg et al [20] examined patterns in the consumption of health-related videos on social media among urban and rural populations and explored how this consumption may be linked to awareness of specific health topics. They found that 59.6% of US adults watched health videos, with younger adults and college graduates being more likely to do so [20]. However, our study is the first to report associations between watching health-related videos and perceived discrimination, subjective numeracy, and interpersonal communication, thereby highlighting the unique ways these factors may influence health information–seeking behaviors on social media.

Other research has highlighted that younger adults are more likely to use social media for health information–seeking purposes, often engaging with content passively or actively seeking it out through groups and pages [21]. Our findings are consistent with prior findings that demonstrate higher engagement on social media among younger adults. According to the Pew Research Center, there are racial/ethnic differences in social media use, with Hispanic users having the highest use across platforms [1]. Similarly, in our study, the higher engagement among Black and Hispanic individuals may reflect a broader trend of these groups using social media to access health information, possibly due to perceived gaps in traditional health care services, disparities in health insurance coverage, and limited access to high-quality health education.

Our study also sheds light on the novel finding that perceived racial discrimination is significantly associated with increased viewing of health-related videos on social media. This suggests that individuals who feel marginalized by health care systems may turn to social media as an alternative source of health information; this finding warrants further exploration. Additionally, participants who reported talking about health with friends or family had significantly higher odds of watching health-related videos on social media than those who did not, suggesting that interpersonal health discussions may encourage individuals to engage in digital health information–seeking and facilitate the cross-platform circulation of health content within social networks. Finally, our results indicate that individuals with higher subjective numeracy (ie, those who self-report understanding medical statistics with ease) and educational attainment are more likely to watch health-related videos. This may be because these individuals are better equipped to search for and interpret complex health information (ie, they may have higher health literacy).

### Implications for Practice

These findings have important implications for public health professionals, clinicians, and policymakers. Understanding the demographics and behaviors of those who seek health information on social media can help in designing targeted interventions and communication strategies. For example, health care providers can guide patients toward credible online resources [3,22]. Additionally, efforts to improve social media literacy among the general population could enhance the quality of health information consumed. For example, Polanco-Levicán et al [23] conducted a systematic review analyzing definitions and competencies related to social media literacy. Their findings emphasized critical skills such as evaluating the reliability of information, understanding the context of social interactions on digital platforms, and managing privacy settings—all crucial for discerning credible health information [24]. Notably, the National Academy of Medicine and the World Health Organization have created guidelines for identifying credible sources on social media, which can be integrated into patient education initiatives and ongoing communication training for clinicians [24].

### Strengths, Limitations, and Future Directions

This study used a nationally representative dataset, which included a comprehensive range of health- and technology-related variables. Despite these strengths, several limitations must be acknowledged. First, the data do not specify the type of health videos watched. Second, we do not know how, if at all, watching a video changed behavior or influenced specific medical decision-making. Third, the potential sharing of these videos among friends and family, an important aspect of interpersonal communication, remains unexplored. Finally, because we merged the 5 original frequency categories into a single “ever watched” measure, we could not evaluate whether viewing frequency varied with the factors under study. Future research should further evaluate social media content sharing within one’s interpersonal communication network and the impact of health-related video content on viewer behavior and decision-making processes.

### Conclusion

This study highlights the roles of various sociodemographic and health-related factors on watching health-related videos on social media platforms. By understanding social media engagement patterns, health professionals can design targeted health communication interventions on social media while dually enhancing health and social media literacy.

## Abbreviations

HINTS: Health Information National Trends Survey
IRB: institutional review board
OR: odds ratio
SAS: Statistical Analysis System
